# Representation of Women in Atrial Fibrillation Clinical Trials: A Systematic Review

**DOI:** 10.31083/RCM47907

**Published:** 2026-03-19

**Authors:** Ramzi Ibrahim, Hoang Nhat Pham, Christopher Kanaan, Buthainah Alhwarat, Enkhtsogt Sainbayar, Sabrina Soin, Chadi Ayoub, Alaide Chieffo, Garima Sharma, Jo Protheroe, Justin Z Lee, Reza Arsanjani, Kwan Lee, Mamas A. Mamas

**Affiliations:** ^1^Department of Cardiovascular Medicine, Mayo Clinic, Phoenix, AZ 85054, USA; ^2^Department of Medicine, University of Arizona, Tucson, AZ 85721, USA; ^3^Department of Medicine, University of Arkansas, Little Rock, AR 72205, USA; ^4^Università Vita-Salute San Raffaele, 20132 Milan, Italy; ^5^Inova Schar Heart and Vascular, Inova Health System, Falls Church, VA 22042, USA; ^6^Research Department of Primary Care and Population Health, University College London, W1T 6QR London, UK; ^7^Department of Cardiovascular Medicine, Cleveland Clinic, Cleveland, OH 44195, USA; ^8^Keele Cardiovascular Research Group, Keele University, ST5 5BG Newcastle, UK

**Keywords:** atrial fibrillation, sex disparities, equity

## Abstract

**Background:**

Disparities exist in the representation of genders in cardiovascular clinical trials. Atrial fibrillation (AF) is associated with significant morbidity and mortality; however, understanding regarding the representation of women in AF-related clinical trials remains limited. Therefore, this systematic review sought to evaluate the representation of women in AF-related clinical trials.

**Methods:**

We conducted a systematic review of clinical trials using the PubMed, Scopus, and EMBASE databases from 1996 to January 1st, 2024, focusing on AF-related lifestyle interventions, pharmacological treatments, catheter ablation, and device therapies for AF. Data extraction and analysis encompassed trial characteristics, participant demographics, and funding sources. The primary outcome was the prevalence of female enrollees, quantified through participation-to-prevalence ratios (PPRs). This was estimated overall and stratified by funding source, intervention type, and enrollment region.

**Results:**

Of the 103 clinical trials involving 218,322 participants (39.5% female), the PPR ranged from 0.00 to 1.73, with an average PPR of 1.03. Meanwhile, 43% of the trials exhibited female under-representation (PPR, <0.8). University-funded trials showed higher female enrollment (mean PPR, 0.951) compared to industry/government-funded trials (mean PPR, 0.800). No differences were observed in the representation of women when comparing enrollment regions or intervention types.

**Conclusions:**

Despite advancements in AF management, gender disparities persist in AF-related clinical trial representation, particularly in industry/government-funded studies compared to university-funded trials. Thus, addressing implicit biases and enforcing sex equality guidelines are critical steps toward more inclusive cardiovascular research.

## 1. Introduction

Atrial fibrillation (AF) is the most prevalent arrhythmia clinically, associated with a significant morbidity and mortality burden [1]. AF management has evolved significantly, with progress in predicting complications and improving outcomes related to bleeding and stroke, as well as advancements in pharmacological treatments and ablation techniques [2, 3]. Despite these advances, the burden of AF in women is significant and includes a higher stroke risk, underuse of pharmacotherapies such as anticoagulation, and a propensity for adverse events [4, 5]. Recognizing the pattern of underrepresentation of women in cardiology trials broadly [6, 7, 8, 9], our study specifically evaluated their representation in AF-related clinical trials.

## 2. Methods

We used the Preferred Reporting Items for Systematic reviews and Meta-Analyses (PRISMA) guidelines to structure our review. Given the use of publicly available data during our repository search, ethical board approval was not needed. Article selection began with title and abstract screening via Covidence, a specialized software for systematic reviews (https://www.covidence.org). We conducted the search for clinical trials pertinent to AF on databases including PubMed, Scopus, and EMBASE, covering all records from 1996 until January 1st, 2024. Search terms included “atrial fibrillation” OR “AF” OR “Afib” AND “clinical trial” OR “randomized” OR “controlled” OR “placebo”. The screening process was completed by two independent authors, with a third author available to resolve any uncertainties. After the preliminary title and abstract review, full texts were obtained for potentially relevant studies. The aim was to assess the prevalence of female enrollees in AF-related clinical trials that evaluated interventions for AF. Inclusion criteria were all AF clinical trials that explored the effects of lifestyle interventions, pharmacological treatments, catheter ablation, or device therapies. Exclusion criteria included non-peer-reviewed articles, studies not in English, and books.

We recorded details from each included trial including the journal of publication, the journal’s 2021 impact factor, number of female participants, type of intervention (i.e., lifestyle, pharmacological, catheter ablation, device therapies), statistical significance of primary endpoints, funding sources as categorized on ClinicalTrials.gov (i.e., government, industry, university), and geographical location of trial enrollment (i.e., North America, Europe, Asia, Australia, and South America). Because the goal was to evaluate representation of women in AF intervention trials broadly, we did not stratify trials by underlying disease etiology (e.g., rheumatic vs. non-rheumatic AF).

Descriptive statistics are presented as means or percentages. Trial participation by women was quantified using participation-to-prevalence ratios (PPRs), a method similar to that used by the Food and Drug Administration (FDA). PPR was calculated at the trial level by dividing the percentage of women enrolled in each trial by the estimated global proportion of women living with AF. We referenced the most recent worldwide epidemiology study of AF using the 2010 Global Burden of Disease Study to estimate the global incidence of AF among women [10]. A PPR below 0.8 indicated under-representation, above 1.2 indicated over-representation, and a PPR between 0.8 and 1.2 signified a representative sample. We compared mean PPRs across categories: operative (catheter ablation, devices) versus non-operative interventions (pharmacological, lifestyle), funding sources (university versus industry/government), and regional enrollment using unpaired *t*-tests and Tukey’s Honest Significant Difference post-hoc tests. Additionally, we examined average PPR trends from the earliest to the latest years of publication using a linear regression model, yielding a beta-coefficient. Temporal trends were assessed using linear regression with trial-level PPR as the dependent variable and publication year as the independent variable. Models were unweighted and did not cluster by study because enrollment sizes and intervention types were heterogeneous. A two-tailed *p*-value of <0.05 was deemed statistically significant. Statistical analysis was completed using Stata (StataCorp LLC - College Station, TX, USA).

## 3. Results

**Fig. 1.**
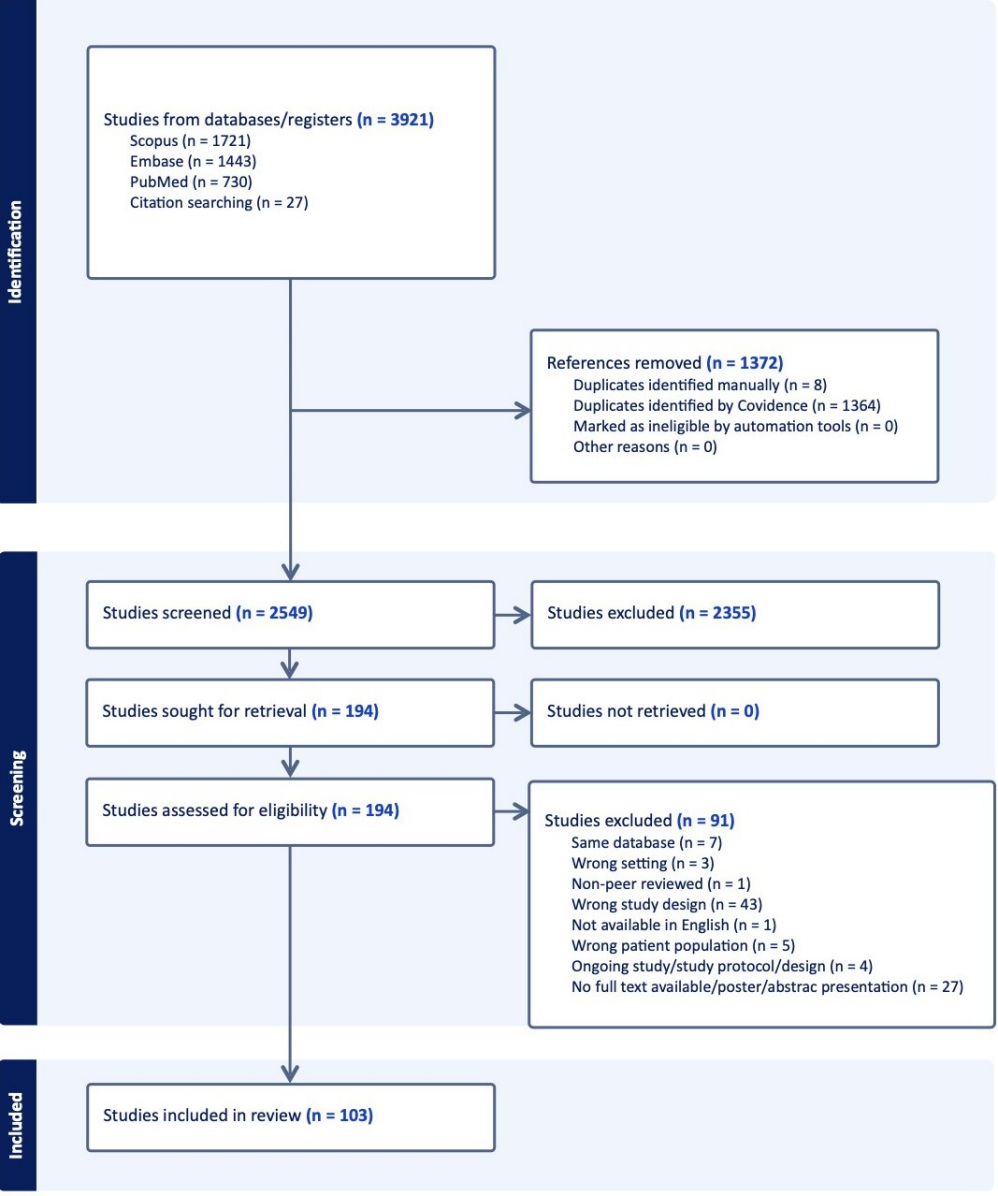
**Preferred Reporting Items for Systematic reviews and Meta-Analyses (PRISMA) diagram**. Systematic approach to inclusion of appropriate trials. PRISMA flow diagram illustrating the identification, screening, eligibility assessment, and inclusion of atrial fibrillation clinical trials published between 1996 and 2023. Reasons for exclusion at each stage are detailed according to PRISMA guidelines.

**Fig. 2.**
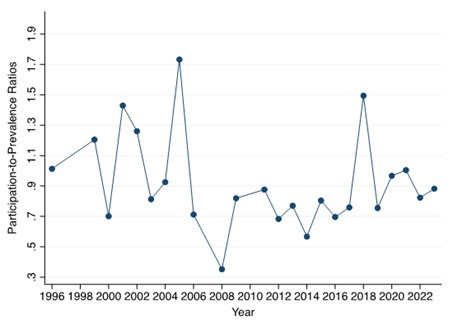
**Temporal changes in participation-to-prevalence ratios (PPRs)**. Scatter plot and fitted linear regression line evaluating temporal trends in trial-level PPR from 1999 to 2023. Each point represents the mean PPR across trials for that year. The regression model used publication year as the independent variable and trial-level PPR as the dependent variable (β = –0.002; 95% CI –0.012 to 0.008; *p* = 0.703). No smoothing beyond the linear fit was applied.

A total of 103 clinical trials comprising 218,322 patients (39.5% female) were included in the final analysis (Table 1 and Fig. 1). All trials were published between 1996 and 2023, with the sample size ranging from 26 to 47,333 participants. There was no significant change in average PPR from 1999 to 2023 (β_trend_ = –0.002, *p* = 0.703) (Fig. 2). The trials were characterized as exploring the impact of pharmacological therapy (42.7%), education (8.7%), lifestyle (5.8%), and operative intervention (60.2%). The majority of trials were funded by government/industry (68.0%), followed by university funding (32.0%).

**Table 1.** **Study Characteristics**.

Variable	Description
Number of trials	103
Number of women enrollees (%)	86,303 (39.5%)
Types of intervention (%)	Pharmacological: 44 (42.7%)
	Education: 9 (8.7%)
	Lifestyle: 6 (5.8%)
	Device: 62 (60.2%)
Funding sources (%)	Government/industry: (68.0%)
	University funding: (32.0%)
Primary outcome indicated as significant (55.0%)	57 trials (55.0%)

Characteristics of the included studies in our analysis. Values represent counts or percentages of trials unless otherwise specified. Intervention categories were defined as follows: operative interventions included catheter ablation and device-based therapies; non-operative interventions included pharmacologic, lifestyle, and education-based strategies. Funding source classifications were obtained from ClinicalTrials.gov or the published manuscript when available. Percentages may not sum to 100% due to rounding and overlap/>1 grouping in trials.

Among all trials, the per-trial percentage of females enrolled ranged from 0% to 66.7% and the per-trial PPR estimates ranged from 0.00 to 1.73, with an overall PPR of all trials estimated at 1.03. 44 trials (43%) had PPR under 0.8, reflecting female under-representation. Although a total of 57 trials (55%) had statistically significant primary outcomes, only 6 trials (6%) described gender-specific outcomes.

Compared to the mean PPR in industry/government-funded clinical trials (0.800), the mean PPR in university-funded clinical trials was higher (0.951), indicative of increased enrollment of women in university-funded clinical trials (Table 2). However, the mean PPR was not different between operative (0.824) and non-operative interventions (0.881) in the clinical trials or across the three different enrollment locations.

**Table 2.** **PPR comparisons**.

Variable	Groups compared	Number of trials	Average PPR	95% CI	*p*-value
Funding source	Industry & government	70/103	0.800	0.741–0.859	0.027*
	University	49/103	0.951	0.881–1.021	
Intervention type	Operative	62/103	0.824	0.762–0.886	0.355
	Non-operative	59/103	0.881	0.817–0.945	
Enrollment region	North America	21/103	0.903	0.796–1.010	0.708
	European	36/103	0.857	0.775–0.939	
	Multi-regional	46/103	0.834	0.762–0.906	
Temporal trends in PPR from 1996 to 2023	β-coefficient = –0.002				0.703

Estimated PPR among trial categories, including operative versus non-operative, funding source, and enrollment region. * statistical significance (*p* < 0.05). PPR was calculated at the trial level by dividing the percentage of female trial participants by the global age-standardized prevalence of atrial fibrillation in women. Funding source/intervention type/regional categories were not always mutually exclusive, resulting in an overlap/>1 grouping in trials. *p*-values reflect two-sided comparisons; statistical significance was defined as *p* < 0.05.

## 4. Discussion

Our review highlights a persistent under-representation of women in AF-related clinical trials. Despite efforts to enhance inclusion, women accounted for only 39.5% of participants across 103 trials. 43% of the total trials had a PPR below 0.8, revealing a significant gender gap. This was more pronounced in industry and government-funded clinical trials compared to those funded by universities. These results represent systemic inequality issues in the realm of cardiovascular research, where scientific accuracy directly informs patient care.

Historical barriers, such as the FDA’s prior constraints on enrolling pre-menopausal women and the delay in embracing the National Institutes of Health’s (NIH) inclusivity guidelines, have had a lasting impact on cardiovascular research [11, 12]. Consequently, women are underrepresented in cardiovascular clinical trials [8]. This under-representation spans various areas of investigation, including heart failure, device implantation, and coronary artery disease [6, 7, 8, 9]. Studies have also shown that trials related to arrhythmia are less likely to include women, who are more prone to adverse events and have higher recurrence rates of AF following procedural interventions [13]. In our study, only 6% of trials reported sex-stratified outcomes, highlighting a major disconnect between enrollment and analytic equity. Representation alone does not ensure meaningful evaluation of sex-specific efficacy or safety. This lack of reporting perpetuates uncertainty regarding how AF therapies, including ablation strategies, antiarrhythmic medications, and rate-control agents, perform differently in women.

The presence of gender-based differences in cardiovascular care emphasizes the importance of gender-specific evaluations in pathogenesis, prevention, and treatment outcomes. Women experience adverse reactions to cardiovascular medications more frequently than men. Furthermore, women who do participate in trials are often older, bearing a heavier comorbidity load, including conditions like hypertension, heart failure, and valvular heart disease [13, 14, 15]. Additionally, women are at a greater risk of stroke in the setting of AF, which tends to be more severe [4, 5]. There is also a concerning trend where female patients with AF are less frequently prescribed anticoagulants and are less likely to be referred for ablation therapies, a disparity that highlights the critical need for more equitable research practices [13]. Similarly, multiple factors may explain why university-funded trials demonstrated higher PPRs than industry/government-funded trials. University-based studies often recruit from academic centers with established community outreach, broader referral networks, and greater emphasis on equitable enrollment practices. Industry and government-funded trials frequently prioritize rapid enrollment, procedural interventions, or select high-volume centers, settings where women may be less frequently referred or may decline participation due to risk perception, comorbidity profile, or caregiving responsibilities. These structural differences likely contribute to institutional variation in female representation.

Although the average PPR was close to 1.0, this figure masks heterogeneity: nearly half of all trials had PPR <0.8. A small number of trials with disproportionately high female enrollment increased the overall mean but did not reflect the distribution across studies. Thus, adequate representation on average coexisted with widespread under-representation at the individual-trial level. Multiple reasons may explain the frequent under-representation of women in AF related clinical trials. Regulatory bodies, including the FDA, should enforce stricter guidelines to enable more equitable representation of women participants in clinical trials. Recruitment strategies should also entail greater efforts to target women participants. Essential to these processes are the education and training of clinical trialists, researchers, and physicians to recognize and mitigate potential implicit biases that can skew recruitment. An equally important measure is the rigorous reporting of gender-based outcomes, which will yield a more thorough assessment of treatment efficacy and safety. Under-representation and limited sex-specific reporting may contribute to persistent uncertainty regarding the comparative effectiveness of AF therapies in women. Prior studies suggest sex-based variation in ablation success, bleeding risk from anticoagulation, and differential pharmacodynamic responses, yet these differences cannot be adequately evaluated without purposeful inclusion and analysis.

### Limitations

Our analysis includes limitations. The application of using the PPR to measure representativeness of women in AF-related trials relies on the accuracy of the prevalence data used. We relied on Global Burden of Disease data, given that this is the most recent representation of AF global estimates. Moreover, trials predominantly originated from North America and Europe, with fewer from regions where rheumatic AF is more common. Thus, our findings may not fully reflect sex-based representation patterns in areas with different AF etiologies. Additionally, because we restricted our search to English-language publications, AF trials conducted in non-English-speaking regions may be underrepresented, potentially influencing observed geographic and funding-related patterns. Lastly, our statistical limitations warrant cautious interpretation. Our mean PPR comparisons used *t*-tests, whereas PPR distributions may deviate from normality. Non-parametric methods yielded similar qualitative conclusions, but this remains a methodological limitation. Temporal analyses used a simple linear model, which may not capture non-linear or period-specific variations in PPR. More advanced modeling (e.g., splines) was not feasible due to limited numbers of trials in earlier decades.

## 5. Conclusions

Our systematic review revealed significant disparities regarding gender balance in AF clinical trials. Our findings also indicated a lesser representation of women in trials funded by industry and government sources. These results highlight the critical need for the implementation of updated guidelines that rigorously enforce gender equality in clinical research.

## Availability of Data and Materials

All data used in this study were from published literature and is therefore publicly available.

## Supplementary Material

Supplementary material associated with this article can be found, in the online version, at https://doi.org/10.31083/RCM47907.
